# Strangulated Femoral Hernia Turned to Be Peritoneal Cyst

**DOI:** 10.1155/2012/528780

**Published:** 2012-11-19

**Authors:** Dionysios Dellaportas, George Polymeneas, Christina Dastamani, Evi Kairi-Vasilatou, Ioannis Papaconstantinou

**Affiliations:** ^1^2nd Department of Surgery, University of Athens, Aretaieion Hospital, 76 Vas. Sofias Avenue, 115 28 Athens, Greece; ^2^Department of Pathology, University of Athens, Aretaieion Hospital, 76 Vas. Sofias Avenue, 115 28 Athens, Greece

## Abstract

*Introduction*. A peritoneal inclusion cyst is a very rare mesenteric cyst of mesothelial origin usually asymptomatic. A rare case of an 82-year-old white Caucasian female with a femoral hernia containing a large peritoneal inclusion cyst, mimicking strangulated hernia, is presented herein. *Case Presentation*. The patient was admitted to our hospital suffering from a palpable groin mass on the right, which became painful and caused great discomfort for the last hours. Physical examination revealed a tender and tense, irreducible groin mass. An inguinal operative approach was selected and the mass was found protruding through the femoral ring. After careful dissection it turned out to be a large unilocular cyst, containing serous fluid, probably originating from the peritoneum. McVay procedure was used to reapproximate the femoral ring. Histologic examination showed a peritoneal inclusion cyst. *Discussion*. Peritoneal inclusion cysts are usually asymptomatic but occasionally present with various, nonspecific symptoms according to their size. Our case highlights that high index of clinical suspicion and careful exploration during repair of a hernia is mandatory in order to reach the correct diagnosis about hernia's contents.

## 1. Introduction

A peritoneal inclusion cyst is a very rare mesenteric cyst of mesothelial origin occurring in the peritoneal cavity, mostly affecting women in the reproductive age. Unilocular peritoneal inclusion cysts are usually asymptomatic, but occasionally present with various, nonspecific symptoms, which makes correct preoperative diagnosis difficult [1]. They may be attached or lie free in the peritoneal cavity, and occasionally they may involve the round ligament simulating an inguinal hernia. Femoral hernias comprise 6–17% of abdominal wall hernias and usually contain abdominal viscera [2]. We present a rare case of an 82-year-old white female with a femoral hernia containing a large peritoneal inclusion cyst, mimicking strangulated hernia.

## 2. Case Presentation

**Figure 1:**
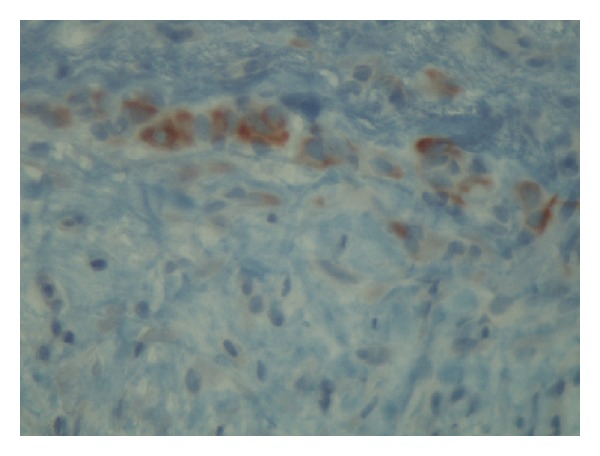
Histological section of the cystic wall showing the presence of mesothelial cells (Hematoxylin-Eosix ×400).

**Figure 2:**
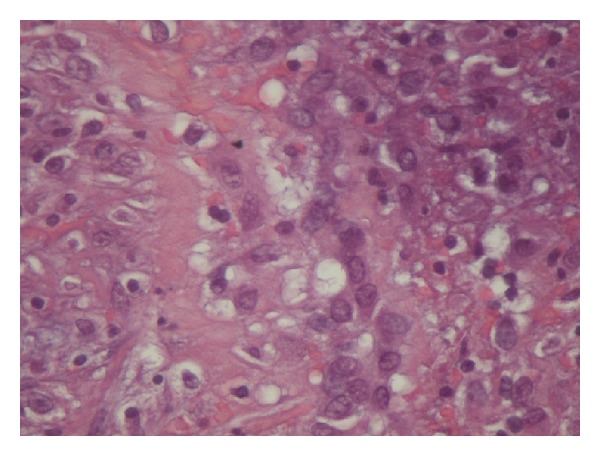
Positive immunohistochemical stain for CK 5/6 confirming the presence of the above mesothelial cells (×400).

An 82-year-old white Caucasian female was admitted to our hospital suffering from a palpable groin mass on the right, which appeared two days ago, but for the last hours before admission it became painful and caused great discomfort. The mass was tender and tense on physical examination measuring at least 8 cm on diameter, and it was not reducible. The initial differential diagnosis was either a strangulated femoral or groin hernia. She had no history of other hernias and her laboratory findings were within normal range. Also no history of previous surgery, endometriosis, or pelvic inflammatory disease existed. An inguinal approach was selected and after opening the skin and subcutaneous fat the mass was found protruding through the femoral ring. Our initial thought was that it was a bulge of abdominal contents covered by peritoneum, but after careful dissection it turned out to be a large unilocular cyst, containing serous fluid, probably originating from the peritoneum. Frozen section was negative for malignancy and cyst excision was easily performed. McVay procedure was used to reapproximate the femoral ring. The patient's postoperative course was uneventful, and she was discharged on the first postoperative day. The histologic examination of the cyst showed a fibrous wall of variable thickness with inflammatory and hemorrhagic infiltrations. Its inner surface was almost completely covered with hemorrhagic tissue and fibrin, lacking epithelial lining. Focally, the presence of a few mesothelial cells in a linear pattern was noted (Figure 1). The immunohistochemistry [Vimentin (+), CK5/6 (+)] confirmed the mesothelial origin of these cells (Figure 2). Physical examination and ultrasonography on follow-up visit after three months showed no recurrence of the hernia or the cyst. 

## 3. Discussion

Peritoneal inclusion cysts are very rare and only about 900 cases are reported in the literature [3]. Those cysts are usually asymptomatic but occasionally present with various, nonspecific symptoms according to their size mostly, which may reach 40 cm in diameter [1]. Acute abdomen due to complications including rupture, obstruction, inflammation, infection, torsion, or hemorrhage within the cyst or, more rarely, ascites, may also be present. Current theory for the formation of these cysts indicates that they are the result of the congenital incomplete fusion of the mesothelial-lined peritoneal surfaces and affect predominantly women in the reproductive age group. Some investigators have mentioned that peritoneal inclusion cysts may form as a result of a localized peritoneal fluid collection due to the presence of peritoneal adhesions, and these peritoneal adhesions have a relation to previous pelvic surgery, endometriosis, or pelvic inflammatory disease [4]. Peritoneal cysts can been classified according to the de Perrot classification based on their cellular origin (1-lymphatic, 2-Mesothelial, 3-enteric, 4-Urogenital, 5-Mature cystic teratoma, 6-Non pancreatic pseudocysts) [5]. Peritoneal inclusion cysts are unilocular or multilocular cystic lesions lined by flat cuboidal mesothelial cells and their wall is fibrotic, lacking any lymphatics or muscular structures [6]. Cross-sectional imaging modalities help us to diagnose these cysts, as long as laboratory values are unremarkable and laparoscopy or laparotomy is rarely necessary in order to characterize promptly the lesion. Observation with serial imaging is a feasible management option for asymptomatic patients with an incidentally discovered peritoneal inclusion cyst. Treatment, when complications arise is complete surgical excision open or laparoscopically. Cyst puncture, simple drainage, and marsupialization are treatment options that should not be performed due to their low efficacy and high risk of complications [7]. 

A femoral hernia usually presents as a tender, nonreducible swelling, situated below, and lateral to the pubic tubercle. The differential diagnoses include inguinal hernia, lipoma, saphena varix, enlarged lymph nodes, femoral artery aneurysm, sarcoma, obturator hernia, psoas abscess, psoas bursa, and in males, ectopic testis. Different contents in femoral hernias have been reported in the literature, such as appendix, small intestine, omentum, bladder, Meckel's diverticulum, ectopic testis, adnexa, and stomach [2]. Whenever diagnostic difficulties are encountered imaging modalities as ultrasound of the abdomen and groins seem to provide sensitive data for distinguishing between congenital abnormalities, noncongenital hernias, vascular conditions, infectious or inflammatory processes, and neoplasms. Sonography can diagnose and differentiate between various inguinal region hernias [8, 9]. Also, peritoneal cyst protruding through the femoral ring and mimicking a femoral hernia is reported before [10]. Our case was similar to the last case and was operated emergently turning out to be a simple peritoneal cyst.

In conclusion, high index of clinical suspicion and careful exploration during repair of a femoral hernia is mandatory in order to reach the correct diagnosis about the contents of the hernia.
